# Intersex health training is associated with sustained competency gains in the healthcare workforce

**DOI:** 10.3389/fpubh.2026.1809092

**Published:** 2026-06-29

**Authors:** Ughetta Maria Favazzi, Angela Ruocco, Martina Manoli, Marta Borgi, Rosilde Di Pirchio, Giorgio Malorni, Pietro Carbone, Debora Guerrera, Francesca Molinaro, Federica Maria Regini, Andrea Vittozzi, Maria Cristina Meriggiola, Jiska Ristori, Alessandra Daphne Fisher, Alfonso Mazzaccara, Elena Ortona, Matteo Marconi, Marina Pierdominici

**Affiliations:** 1 Training Office, Istituto Superiore di Sanità, Rome, Italy; 2 Reference Centre for Gender Medicine, Istituto Superiore di Sanità, Rome, Italy; 3 Department of Medical and Surgical Sciences and Advanced Technologies “GF Ingrassia”, University of Catania, Catania, Italy; 4 Division of Gynecology and Human Reproduction Physiopathology, IRCCS Azienda Ospedaliero-Universitaria di Bologna, Bologna, Italy; 5 Department of Medical and Surgical Sciences (DIMEC), University of Bologna, Bologna, Italy; 6 Cognitive Behavioral Therapy Centre, Florence, Italy; 7 Endocrinology Unit, Department of Multidimensional Medicine, Azienda USL Toscana Centro, Florence, Italy; 8 National Centre for Clinical Governance and Excellence in Care, Istituto Superiore di Sanità, Rome, Italy

**Keywords:** e-learning, healthcare workforce, intersex health, longitudinal studies, professional competence, public health education

## Abstract

**Introduction:**

Education on intersex health remains limited across healthcare professional training pathways, resulting in persistent gaps in workforce competencies. Existing educational initiatives are often fragmented, small in scale, and rarely evaluated beyond immediate post-training outcomes. Robust evidence on the sustainability of educational effects over time is still scarce.

**Objectives:**

To evaluate the immediate and six-month educational impact of a large-scale e-learning training program on intersex health among healthcare professionals.

**Methods:**

We conducted a longitudinal cohort study of a national asynchronous e-learning program delivered between February and August 2023. The program targeted physicians, psychologists, midwives, and healthcare assistants and was designed using a problem-based learning approach. Educational outcomes were assessed at baseline (T0), immediately after course completion (T1), and at six-month follow-up (T2), using self-reported measures of attitudes and perceived skills, a knowledge assessment test, and a training satisfaction questionnaire. Statistical analyses included paired t-tests, ANCOVA, and McNemar tests.

**Results:**

Among 6,332 participants completing both T0 and T1 assessments, 1,036 also completed the six-month follow-up. Participants were predominantly female (73.9%), with psychologists (47.8%) and physicians (39.1%) representing the largest professional groups. Significant improvements were observed at T1 in attitudes (+0.54), perceived skills (+1.45), and knowledge (+11.7 percentage points; all *p* < 0.001). Improvements were largely sustained at T2, with only modest declines across domains. Baseline differences by gender, geographic area, and professional group were substantially reduced following training. Overall satisfaction with the program was high (mean score 4.48/5) across participant subgroups.

**Conclusion:**

This study provides longitudinal evidence that a large-scale e-learning training program was associated with significant and sustained improvements in healthcare workforce competencies in a traditionally under-addressed area of care. Structured, cross-disciplinary training delivered through accessible digital formats may offer a scalable strategy to strengthen workforce preparedness and reduce educational gaps in intersex healthcare.

## 1 Introduction

Health inequities affecting minoritized populations are increasingly recognised as a priority within health professions education and public health agendas in Europe. Intersex individuals - an umbrella term for people born with sex characteristics (such as genitals, gonads, chromosomes, or hormones) that do not fit typical binary definitions of male or female bodies - remain among the most underserved populations, facing systemic discrimination and persistent barriers to safe, respectful, and evidence-informed health care (1–4). Despite growing attention to human rights and ethical standards, substantial gaps persist in the education and training of healthcare professionals on the psychosocial, clinical, legal, and ethical dimensions of intersex health, limiting the delivery of appropriate and equitable care.

In clinical contexts, intersex individuals are often described as having differences or disorders of sex development (DSDs), a heterogeneous group of congenital conditions involving atypical chromosomal, gonadal, or anatomical development (5, 6). In legal and human rights frameworks, the term variations of sex characteristics (VSCs) is increasingly adopted to emphasise biological diversity and reduce pathologizing language (7). In this article, we use the terms intersex, DSDs, and VSCs interchangeably, reflecting both clinical and inclusive international terminology.

These conditions may be identified at birth due to atypical genital anatomy, during childhood or adolescence, or later in life for example in the context of delayed puberty or infertility (8). While some forms, such as salt-wasting congenital adrenal hyperplasia, require timely medical intervention, many are not life-threatening yet have historically been managed through irreversible clinical practices in the absence of robust evidence on long-term outcomes. The 2006 Chicago Consensus advocated a multidisciplinary, patient-centred approach grounded in shared decision-making and psychosocial support (5). However, uncertainties remain regarding gender development, fertility preservation, and the timing and appropriateness of early medical and surgical interventions (6, 8).

The lack of systematic and structured training on intersex health within undergraduate, postgraduate, and continuing medical education pathways continues to represent a major barrier to the implementation of inclusive, evidence-based care (2–4, 9–16). Intersex-related content is frequently subsumed within broader LGBT+ cultural competence training, where the specific psychosocial, clinical, legal, and ethical complexities of intersex care are often insufficiently addressed (17–22). Existing educational initiatives are typically fragmented, limited in scale, discipline-specific, and evaluated only immediately after delivery, providing little evidence on the sustainability of learning outcomes or their relevance across different professional groups (23–26).

To address these gaps, the Istituto Superiore di Sanità (ISS; National Institute of Health, Italy), in collaboration with the Ufficio Nazionale Antidiscriminazioni Razziali (UNAR; National Office against Racial Discrimination), developed the first publicly led national training program in Europe dedicated exclusively to intersex health. Delivered through the ISS asynchronous e-learning platform, the program adopted a multidisciplinary approach and targeted physicians, psychologists, midwives, and healthcare assistants nationwide, embedding intersex-specific competencies within the formal continuing medical education system.

This study evaluates the longitudinal educational impact of this program, moving beyond immediate post-training assessment to examine the sustainability of learning outcomes over time. By analysing changes in attitudes, perceived skills, and knowledge across professional groups, the study addresses a critical gap in the medical education and health workforce literature. The findings provide empirically grounded insights into how large-scale, institutionally governed continuing education interventions can strengthen workforce preparedness in neglected areas of care and offer a replicable model for integrating intersex-specific content into formal professional training pathways.

## 2 Materials and methods

### 2.1 Course design, learning methodology, and participant overview

We conducted a longitudinal cohort study evaluating the educational impact of a national, institutionally delivered e-learning course on intersex health. The distance-learning course “Intersex individuals: health, bioethics, and legal perspectives” was freely offered through the ISS e-learning platform (EDUISS, www.eduiss.it) and delivered entirely in Italian. The course employed a Problem-Based Learning (PBL) framework, grounded in adult learning principles that fosters active, self-directed learning by engaging participants in solving real-world problems relevant to their professional practice (27). The PBL approach was adapted for an asynchronous e-learning environment, enabling participants to independently complete a structured seven-step cycle supported by interactive tools such as forums, quizzes, and curated resource materials. This methodology had been successfully applied in previous ISS distance-learning courses, demonstrating its effectiveness in enhancing professional knowledge, attitudes, and competencies (28).

A multidisciplinary scientific board, composed of healthcare professionals, legal scholars, and bioethicists, developed the curriculum for this competency-oriented, publicly governed training program. Individuals with lived experience of intersex variations were not directly involved in the development of the training curriculum, which may represent a limitation. However, the program is part of a broader institutional initiative that includes ongoing collaboration with intersex civil society organizations.

The course was structured around five Learning Objectives (LOs), covering key domains of intersex health (see Supplementary material Table S1 for a detailed overview of module content).

LO1: Components of sexual identity and its biological foundations.
LO2: Psychological and medical aspects of intersex care across the lifespan.
LO3: The health provider–patient relationship and approaches to family support.
LO4: Legal aspects related to intersex variations.
LO5: Ethical considerations in the care of individuals with intersex variations.

The course was designed for physicians, psychologists, midwives, and healthcare assistants and ran from 27 February 2023 to 31 August 2023, with a participant capacity of up to 10,000 healthcare professionals nationwide. Completion granted 16 Continuing Medical Education (CME) credits, corresponding to approximately 16 h of study. A total of 10,000 healthcare professionals enrolled in the course. Of these, 8,361 accessed the learning platform at least once, while 1,627 never logged in. Among active participants, 1,599 did not engage further with the course content, 374 dropped out during training, and 56 did not pass the final assessment. Overall, 6,332 participants successfully completed the course and were included in the present analysis.

The course consisted of four main sections:

*Introductory section*: this included an overview of course structure and objectives, as well as baseline assessments (T0), comprising the Attitudes and Skills Questionnaire (ASQ) and the Knowledge Assessment Test (KAT) (details provided below).

*PBL cycle*: this section delivered the core educational content using the PBL framework. Resources included problem scenarios, a Sharable Content Object Reference Model (SCORM) exercise for problem analysis and learning objective identification, curated research materials (bibliographic references, recommended websites, specialized readings, and expert-led audio-video tutorials), and structured activities for problem resolution.

*Concluding section*: immediately after course completion (T1), participants repeated both the ASQ and the KAT. A final certification exam (consisting of 48 multiple-choice questions, with a 75% passing score and up to three attempts allowed) and a Training Satisfaction Questionnaire (TSQ) were also administered.

*Follow-up section*: 6 months post-course (T2), participants were invited to retake both the ASQ and the KAT, allowing assessment of the sustainability of learning outcomes over time. These were available from 29 February 2024 to 31 March 2024.

### 2.2 Data collection and tools

At enrolment, demographic and professional characteristics, including gender, age, geographic area, and profession, were collected. Two tools were used to establish T0 measures and monitor changes in educational outcomes over time (T1 and T2):

*Attitudes and skills questionnaire (ASQ)*: this purpose-built instrument was developed to align with the learning objectives and intended educational outcomes of the course. It comprised two sections, Attitudes and Skills. Attitudes: five items were presented as statements (e.g., “Biological factors significantly influence the development of gender differences”), each introduced by the prompt “With regard to the care of intersex individuals, I believe that…”. Participants rated their agreement on a 5-point Likert scale (1 = completely disagree to 5 = completely agree), with higher scores indicating more positive attitudes toward intersex care. Skills: six items assessed self-perceived professional competence, introduced by the prompt “With regard to the care of intersex individuals, if given the opportunity, I would be able to…”. Responses were recorded on a 5-point scale (1 = not at all to 5 = completely), with higher scores indicating greater self-reported ability to perform relevant tasks.

*Knowledge Assessment Test (KAT)*: This is a 10-item multiple choice test (two questions per LO, Q1–Q10), designed to evaluate core conceptual knowledge acquisition, aligned with the course objectives. Each correct answer was assigned 1 point, while blank or missing responses were coded as incorrect (0 points), yielding a total possible score ranging from 0 to 10. The full list of knowledge assessment questions is provided in Supplementary Table S2.

*Training satisfaction questionnaire (TSQ)*: questionnaire assessing course satisfaction. The TSQ included 18 items rated on a 5-point Likert scale (1 = strongly disagree to 5 = strongly agree), plus two open-ended questions. Higher scores indicate greater satisfaction with the training.

This was a non-interventional educational study involving healthcare professionals and did not include patients, clinical interventions, biological samples, or identifiable health data. Participation was voluntary, and informed consent for the use of pseudonymized data was obtained from all participants at registration. According to the applicable Italian regulatory framework, ethics committee approval is not required for this type of non-interventional educational research. The study was conducted in compliance with European Union data protection regulations, including Regulation (EU) 2016/679 (General Data Protection Regulation, GDPR).

### 2.3 Statistical analysis

Data were extracted from the EDUISS platform. Descriptive statistics (absolute frequencies, percentages, and means) were used to summarize participants’ demographic and professional characteristics. The psychometric properties of the attitudes and skills sections were evaluated using exploratory factor analysis (principal axis factoring with Promax rotation) at T0, T1, and T2. A two-factor solution was retained based on eigenvalues and interpretability, corresponding to self-perceived skills and attitudes. Internal consistency was assessed using Cronbach’s alpha. Detailed factor analytic results are reported in Supplementary Table S3. Based on the factor structure, two composite indices were created by averaging the six items assessing skills and the five items assessing attitudes, respectively. These indices were computed at T0 and T1 to allow for consistent pre-post educational comparisons.

Paired samples t-tests were used to assess changes from T0 to T1 and from T0 to T2 at the item level; effect sizes were expressed as Cohen’s d. To explore group differences, one-way ANOVAs were done on baseline scores (T0) for each index, and ANCOVAs were used at T1, controlling for baseline levels, to assess whether educational gains varied by gender, age group, profession, and geographic area. Knowledge was assessed as the percentage of correct responses at T0, T1, and T2. Changes in individual knowledge items over time were examined using McNemar tests, comparing paired proportions of correct answers across T0-T1, T2-T0, and T2-T1. Satisfaction ratings were summarized using a composite indicator (Satisfaction Total Index, STI), calculated as the mean of 18 Likert-scale items (Cronbach’s *α* = 0.96). One-way ANOVAs were performed to examine subgroup differences in STI scores by gender, age group, profession, and geographic area. To assess potential attrition bias, baseline characteristics were compared between participants who completed the six-month follow-up and those who did not using chi-square tests for categorical variables.

Given the number of statistical tests performed, no formal correction for multiple comparisons was applied. This decision was based on the exploratory nature of some analyses and the large sample size; results were interpreted considering the magnitude and consistency of effects across outcomes and subgroups.

All statistical analyses were conducted using IBM SPSS Statistics, version 28.0 (IBM Corp). All *p* values were two-sided, and values <0.05 were considered statistically significant.

## 3 Results

### 3.1 Participant characteristics

Among the 6,332 participants who completed both T0 and T1 assessments, most were female (73.9%). The largest age groups were 36–45 years (29.1%) and over 55 years (29.8%). Participants were distributed across all Italian regions, primarily in the Northwest (32.0%) and the Centre (25.5%). Psychologists represented the largest professional group (47.8%), followed by physicians (39.1%), midwives (9.4%), and healthcare assistants (3.7%) (Table 1).

**Table 1:** Characteristics of participants completing baseline (T0) and immediate post-training assessments (T1).

Characteristic	Participants, no. (%) (*n* = 6,332)
Gender	
Male	1,651 (26.1)
Female	4,681 (73.9)
Age (years)	
Up to 35	1,239 (19.6)
36 to 45	1844 (29.1)
46 to 55	1,364 (21.5)
Over 55	1885 (29.8)
Italian region area	
Northwest	2029 (32.0)
Northeast	706 (11.1)
Center	1,614 (25.5)
South	1,255 (19.8)
Islands	714 (11.3)
Foreign	14 (0.2)
Health professions	
Healthcare assistant	232 (3.7)
Midwife	593 (9.4)
Physician	2,477 (39.1)
Psychologist	3,030 (47.8)

At baseline (T0), significant differences in knowledge scores were observed across professional groups (*p* < 0.001) and geographic areas (*p* = 0.045), with psychologists and participants from the Northwest and Northeast scoring higher than other groups. No significant baseline differences in knowledge were found by gender or age group. For self-reported attitudes, women scored higher than men (mean 3.31 vs. 3.02; *p* = 0.017), and psychologists scored higher than healthcare assistants (*p* < 0.001). For self-reported skills, older participants (> 46 years) reported lower baseline scores than younger age groups (*p* = 0.003), and psychologists scored higher than midwives (*p* = 0.004).

### 3.2 Immediate effects of the training (T0 to T1)

#### 3.2.1 Self-reported attitudes

Across the full sample, self-reported attitude scores improved significantly from baseline (mean [SD] 3.30 [1.03]) to immediate post-training (3.84 [1.05]; *p* < 0.001), with a moderate effect size (Cohen’s *d* = 0.51). The largest improvements were seen for bioethical principles (mean change 0.75; *p* < 0.001) and the perceived role of biological factors in gender differences (0.74; *p* < 0.001). More modest but still significant increases were observed for family support resources, psychological support, and legal information (mean changes 0.37–0.47; all *p* < 0.001; Table 2; Supplementary Figure S1a).

**Table 2:** Self-reported attitudes toward intersex care at baseline (T0) and immediate post-training (T1) (*n* = 6,332).

Statement	Mean (SD)		Mean difference T1–T0	*p*-value
	T0	T1		
Biological factors significantly influence the development of gender differences	2.31 (1.21)	3.05 (1.30)	+0.74 (1.47)	<0.001
Psychological support should be provided to intersex individuals during the coming out process	3.68 (1.35)	4.05 (1.24)	+0.37 (1.39)	<0.001
Support resources should be made available to families of individuals diagnosed with an intersex variation	3.65 (1.32)	4.12 (1.21)	+0.47 (1.38)	<0.001
Comprehensive legal information should be made available regarding intersex variations	3.66 (1.29)	4.03 (1.22)	+0.37 (1.34)	<0.001
Bioethical principles relevant to intersex variations must be taken into account	3.22 (1.41)	3.97 (1.26)	+0.75 (1.54)	<0.001

The table reports mean item-level scores among participants (based on a 5-point Likert scale, with higher scores indicating more positive attitudes toward intersex care) and corresponding standard deviations (SDs) for the subset of participants who completed both the baseline (T0) and immediate post-training (T1) assessments (*n* = 6,332). Change scores represent the average per-item difference, calculated as the arithmetic difference between T1 and T0. All *p*-values are from paired t-tests comparing mean scores between T0 and T1. All statements were preceded in the questionnaire by the prompt: “*With regard to the care of intersex individuals, I believe that…*”.

After adjustment for baseline scores, ANCOVA showed no significant sociodemographic differences in post-training attitude scores, indicating consistent attitudinal gains across participant subgroups.

#### 3.2.2 Self-reported skills

The training was associated with significant increases in self-reported skills across all evaluated domains. Overall, mean scores rose from baseline (mean [SD] 2.38 [0.85]) to immediate post-training (3.83 [0.65]; *p* < 0.001), with a large effect size (Cohen’s *d* = 0.94). The largest gains were observed for identifying bioethical issues (+1.70; *p* < 0.001) and understanding legal frameworks (+1.56; *p* < 0.001), followed by psychological and medical aspects of care (+1.42; *p* < 0.001), components of sexual identity (+1.36; *p* < 0.001), family support resources (+1.36; *p* < 0.001), and roles and responsibilities of health professionals (+1.33; *p* < 0.001; Table 3; Supplementary Figure S1b).

**Table 3:** Self-reported skills in intersex care at baseline (T0) and immediate post-training (T1) (*n* = 6,332).

Statement	Mean (SD)		Mean difference T1–T0	*p*-value
	T0	T1		
Describe the components of sexual identity and their biological foundations	2.50 (1.17)	3.86 (0.79)	+1.36 (1.26)	<0.001
Identify relevant psychological and medical factors in the care of intersex individuals	2.46 (1.15)	3.88 (0.74)	+1.42 (1.24)	<0.001
Identify tools and resources to support families of intersex individuals	2.59 (1.19)	3.95 (0.76)	+1.36 (1.27)	<0.001
Define the roles and responsibilities of health professionals in relation to service users	2.63 (1.17)	3.96 (0.71)	+1.33 (1.26)	<0.001
Identify legal frameworks associated with intersex conditions	2.00 (0.99)	3.56 (0.94)	+1.56 (1.26)	<0.001
Identify key bioethical issues related to intersex conditions	2.12 (1.07)	3.82 (0.80)	+1.70 (1.25)	<0.001

The table reports mean item-level scores among participants (based on a 5-point Likert scale, with higher scores indicating improved skills in intersex care) and corresponding standard deviations (SDs) for the subset of participants who completed both the baseline (T0) and immediate post-training (T1) assessments (*n* = 6,332). Change scores represent the average per-item difference, calculated as the arithmetic difference between T1 and T0. All *p*-values are from paired t-tests comparing mean scores between T0 and T1. All statements were preceded in the questionnaire by the prompt: “*With regard to the care of intersex individuals, if given the opportunity, I would be able to*…”.

After controlling for baseline scores, ANCOVA showed no significant sociodemographic differences in post-training skills at T1, indicating homogeneous skill acquisition across participant subgroups.

#### 3.2.3 Knowledge levels

Knowledge levels improved significantly across all assessed items at immediate post-training. Correct response rates increased from baseline (mean [SD] 67.1% [22.6%]) to immediate post-training (78.8% [23.0%]; *p* < 0.001), with a medium effect size (Cohen’s d = 0.52). The largest improvement was observed for legal knowledge related to the sex–gender binary (Q7: +27.1 percentage points; *p* < 0.001). Substantial gains were also observed for components of sexual identity (Q1: +19.2 points; *p* < 0.001), international clinical recommendations for intersex management (Q4: +14.4 points; *p* < 0.001), medical considerations at birth (Q3: +12.5 points; *p* < 0.001), and bioethical issues related to consent for minors (Q9: +11.2 points; *p* < 0.001).

More modest improvements were noted for support for gender identity exploration (Q6: +10.7 points; *p* < 0.001), gender identity definition (Q2: +8.1 points; *p* < 0.001), legal recognition of intersex status (Q8: +6.8 points; *p* < 0.001), medical decision-making principles for minors (Q10: +5.6 points; *p* < 0.001), and ideal health care team composition (Q5: +1.7 points; *p* < 0.001; Table 4; Supplementary Figure S1c).

**Table 4:** Knowledge levels on intersex care by learning objective at baseline (T0) and immediate post-training (T1) (*n* = 6,332).

Learning objective (LO)	Quest.	Correct answers, no. (%)		Difference T1–T0, no. (pp)	*p*-value
		T0	T1		
LO1: Components of sexual identity and its biological foundations	Q1	3,557 (56.2)	4,773 (75.4)	+1,216 (19.2)	<0.001
	Q2	4,587 (72.4)	5,100 (80.5)	+513 (8.1)	<0.001
LO2: Psychological and medical aspects of intersex care across the lifespan	Q3	4,565 (72.1)	5,355 (84.6)	+790 (12.5)	<0.001
	Q4	3,876 (61.2)	4,790 (75.6)	+914 (14.4)	<0.001
LO3: The health provider–patient relationship and approaches to family support	Q5	5,848 (92.4)	5,960 (94.1)	+112 (1.7)	<0.001
	Q6	4,007 (63.3)	4,688 (74.0)	+681 (10.7)	<0.001
LO4: Legal aspects related to intersex variations	Q7	2091 (33.0)	3,808 (60.1)	+1717 (27.1)	<0.001
	Q8	4,182 (66.0)	4,609 (72.8)	+427 (6.8)	<0.001
LO5: Ethical considerations in the care of individuals with intersex variations	Q9	4,578 (72.3)	5,285 (83.5)	+707 (11.2)	<0.001
	Q10	5,191 (82.0)	5,546 (87.6)	+355 (5.6)	<0.001

The table reports item-level data from the Knowledge Assessment Test (Q1–Q10) on the number and percentage of correct answers among participants who completed both the baseline (T0) and immediate post-training (T1) assessments (*n* = 6,332), grouped by Learning Objectives (LOs). Each LO is assessed by two questions; full item wording is provided in Supplementary Table S2. Blank responses were automatically coded as incorrect. Changes (T1–T0) represent the absolute difference in correct response rates between time points, expressed in percentage points (pp). All *p*-values are from McNemar’s test for paired proportions, used to assess the statistical significance of changes in correct responses between T0 and T1.

Although differences in knowledge scores by profession and geographic area were observed at baseline, these were no longer evident in post-training scores, as confirmed by ANCOVA.

#### 3.2.4 Course satisfaction

Overall satisfaction with the training was high, with a mean score of 4.48 (SD 0.56) on a 5-point scale (median 4.67; range 1–5; Supplementary Table S4). Satisfaction scores differed significantly by age group (*p* = 0.011), with higher ratings reported by older participants. No significant differences were observed by gender, professional category, or geographic area (all *p* > 0.05). In open-ended feedback, participants highlighted areas for potential improvement, including greater use of multidisciplinary case discussions and additional opportunities for direct engagement with patients, aimed at further strengthening the practical relevance of the training (data not shown).

### 3.3 Six-month follow-up outcomes (T0, T1, and T2 comparisons)

Among the 1,036 participants who completed the 6-month follow-up (participant characteristics are reported in Supplementary Table S5), attitudes, skills, and knowledge remained significantly higher than baseline, although small but significant declines were observed compared with T1.

To assess potential attrition bias, we compared baseline characteristics between participants who completed T2 and those who did not. No statistically significant differences were observed for gender (*p* = 0.124). Participants who completed T2 were significantly older than non-completers (*p* < 0.001), with a higher proportion of individuals aged over 55 years (44.3% vs. 27.1%) and a lower proportion under 45 years. No statistically significant differences were observed for professional group (*p* = 0.061), although some variation was noted within professional categories, including a lower proportion of midwives among T2 completers (7.1% vs. 9.8%). Statistically significant differences were observed in geographic distribution (*p* = 0.002), with relatively higher representation from Central and Northeast regions and lower representation from Southern regions among T2 completers.

#### 3.3.1 Self-reported attitudes

At 6 months, self-reported attitudes remained significantly higher than baseline (mean [SD] at T2, 3.75 [1.10] vs. 3.33 [1.06]; *p* < 0.001). Compared with immediate post-training, modest but significant declines were seen across most items. The largest reductions were observed for biological factors contributing to gender differences (−0.61; *p* < 0.001) and bioethical principles (−0.45; *p* < 0.001), followed by family support resources (−0.20; *p* < 0.001) and psychological support (−0.12; *p* < 0.005). No significant decline was observed for legal information (−0.07; *p* = 0.112; Supplementary Table S6 and Supplementary Figure S1d).

#### 3.3.2 Self-reported skills

At 6 months, participants continued to report significantly higher skill levels than at baseline (mean [SD] at T2, 3.63 [0.78] vs. 2.40 [0.85]; *p* < 0.001). Compared with immediate post-training, modest but significant declines were observed across all skill items. The largest reductions were observed for legal frameworks (−0.61; *p* < 0.001) and bioethical issues (−0.61; *p* < 0.001), followed by components of sexual identity (−0.44; *p* < 0.001), medical and psychological aspects of care (−0.39; *p* < 0.001), family support resources (−0.32; *p* < 0.001), and roles and responsibilities of health professionals (−0.29; *p* < 0.001; Supplementary Table S7 and Supplementary Figure S1e).

#### 3.3.3 Knowledge levels

At 6 months, knowledge levels remained significantly higher than baseline (mean [SD] at T2, 74.2% [22.8%] vs. 67.1% [22.6%]; *p* < 0.001). Compared with immediate post-training, the largest decline was observed for legal knowledge related to the sex-gender binary (Q7: −23.0 percentage points; *p* < 0.001). Moderate declines were observed for components of sexual identity (Q1: −8.9 points; *p* < 0.001), international clinical recommendations for intersex management (Q4: −8.7 points; *p* < 0.001), support for gender identity exploration (Q6: −7.2 points; *p* < 0.001), and bioethical issues related to consent for minors (Q9: −6.4 points; *p* < 0.001). Smaller but significant declines were noted for medical considerations at birth (Q3: −4.9 points; *p* < 0.001) and legal recognition of intersex status (Q8: −2.6 points; *p* < 0.005). No significant changes were observed for gender identity definition (Q2: −1.6 points; *p* = 0.065), medical decision-making principles for minors (Q10: −0.3 points; *p* = 0.246), or ideal health care team composition (Q5: +0.9 points; *p* = 0.243; Supplementary Table S8 and Supplementary Figure S1f).

## 4 Discussion

This study demonstrates that a large-scale, nationally delivered continuing medical education program focused on intersex health was associated with significant improvements in healthcare professionals’ attitudes, skills, and knowledge, with effects largely sustained at six-month follow-up. Taken together, these findings suggest that a well-designed educational intervention delivered at scale may contribute to meaningful and durable learning gains, particularly when delivered through a structured, cross-disciplinary and institutionally governed approach.

At baseline, we identified disparities in competencies by profession, geography, and gender. Psychologists and health professionals from northern regions demonstrated higher knowledge scores, possibly reflecting greater access to specialized services, training networks, and psychosocial frameworks. Women exhibited more inclusive attitudes, consistent with previous findings on empathy and diversity responsiveness among female health professionals (28–30). Younger participants also reported slightly higher skills, which may reflect greater exposure to contemporary discourses on sex, gender, and patient-centered care. Importantly, these baseline differences were no longer evident following the intervention, indicating the program’s capacity to promote equitable learning gains across heterogeneous professional and sociodemographic groups and to mitigate structural disparities in provider preparedness.

The broad, foundational scope of the program—covering clinical, psychosocial, legal, and bioethical dimensions—proved effective in establishing a shared conceptual framework and minimum competency set across diverse professional profiles. Immediate improvements were strongest in areas historically underrepresented in curricula.

Attitudes improved most for bioethical principles and recognition of biological factors influencing gender differences; skills improved most in identifying bioethical issues and legal frameworks, followed by psychosocial and medical aspects, family support, and professional responsibilities. Knowledge gains were greatest for international clinical recommendations, components of sexual identity, legal frameworks, and bioethical issues related to minor consent (23). Improvements were consistent across professional groups, including psychologists, physicians, midwives, and healthcare assistants, with no evidence of differential effects by profession, suggesting that this foundational training can be delivered effectively across diverse professional profiles.

Our findings extend and strengthen the existing literature on intersex education (23–26). For instance, the DSDnet training school was an important milestone for multidisciplinary collaboration but was limited in scale (87 trainees), relied on subjective outcomes, and lacked longitudinal evaluation (23). Kranenburg et al. (24, 25) showed case-based e-learning could enhance communication skills among pediatric endocrinology fellows, but applicability beyond subspecialty contexts was limited and variability remained high. Gupta et al. (26) demonstrated knowledge and self-efficacy gains in pediatric residents, but with no longitudinal follow-up and limited generalizability. Compared with these previous initiatives, the present study provides evidence from a large, multidisciplinary cohort and demonstrates sustained improvements across multiple competency domains, supporting the feasibility of scalable, workforce-oriented intersex education.

At six-month follow-up, attitudes, skills, and knowledge remained significantly higher than baseline, although modest reductions were observed compared with immediate post-training. Attitudes showed the greatest decrease regarding biological factors and bioethical principles; skills declined in legal and bioethical domains, followed by sexual identity, medical/psychological aspects, family support, and professional roles. Knowledge losses were most evident in legal frameworks and in conceptual domains such as sexual identity, international recommendations, and psychosocial aspects of gender identity exploration. These patterns align with well-established evidence on knowledge retention and decline in adult learning and continuing medical education, including the classic Ebbinghaus forgetting curve model (31), and may reflect the limited reinforcement of legal and conceptual domains in routine clinical practice, highlighting the importance of repeated exposure and application (32, 33).

The six-month follow-up findings should be interpreted in light of the observed attrition pattern. Participants who completed T2 were significantly older and differed in geographic distribution compared with non-completers, suggesting a potential retention bias and possible overestimation of sustained training effects.

Participant feedback highlighted the value of case-based discussion, patient interaction, and multidisciplinary integration—elements consistent with previous educational studies (23). High satisfaction across subgroups suggests broad acceptability of the program, while asynchronous delivery and public sponsorship may have enhanced accessibility, particularly for professionals in underserved or peripheral areas. Together, these findings support digitally delivered training as a potentially practical strategy to improve workforce preparedness where access to specialized education remains uneven.

Several measurement considerations should also be acknowledged. Ceiling effects were observed for some knowledge items, particularly Q5 and Q10, which showed high baseline proportions of correct responses. This may have limited the sensitivity of these items to detect further improvements over time, suggesting that KAT may be less responsive in areas where baseline knowledge is already high. However, these items addressed foundational aspects of intersex care that may already have been relatively familiar to participants. As this represents the first edition of a nationally implemented training program, these findings may inform the refinement of assessment tools and content in future iterations to enhance measurement sensitivity and better capture learning gains across domains.

A further consideration concerns the overlap between items Q9 and Q10 within LO5, as both address dimensions of decision-making for minors, particularly with regard to consent and limits of physician authority. This conceptual proximity may have reduced the discriminative capacity of the LO5 assessment. Future refinements of the KAT may further differentiate these aspects to improve measurement precision.

This study has several strengths. It represents the first publicly led national medical education program on intersex health in Europe, involving a large and diverse professional sample. Additional strengths include the use of structured and pre-tested instruments and a multi-timepoint design that enabled the assessment of changes over time.

Limitations must also be acknowledged. The absence of a control group precludes causal inference; although improvements were observed over time, alternative explanations such as regression to the mean, test–retest familiarity, or natural temporal changes cannot be fully excluded. Outcomes were self-reported and may be influenced by social desirability bias. Direct measures of changes in clinical practice and patient-reported outcomes were not included, limiting the assessment of the training’s impact on real-world practice and patient experience. In addition, some characteristics of the KAT, including ceiling effects and partial overlap between selected items, may have limited its sensitivity to detect changes across domains. Substantial attrition at six-month follow-up may have introduced selection bias and may limit the generalizability of longitudinal findings. Moreover, relatively small subgroup sizes at T2 (e.g., healthcare assistants and midwives) may have reduced statistical power for subgroup comparisons, increasing the risk of Type II error and limiting the ability to detect potentially meaningful differences. No formal correction for multiple comparisons was applied; therefore, the large number of statistical tests performed may increase the risk of Type I error. Finally, as the study was conducted within a single national context, the findings may not be fully generalizable to other settings and may require contextual adaptation across different healthcare and sociocultural environments.

## 5 Conclusion

Our findings have important implications for health workforce development, medical education, and health policy. Public institutions can play a central role in advancing intersex health by embedding structured, evidence-informed training within national education systems. Tiered educational models should be considered, beginning with foundational content and progressing to role-specific modules that include case-based and skills-based components. Future evaluations should incorporate observed practice indicators and patient-reported outcomes to assess long-term clinical impact. Overall, scalable and accessible educational interventions may contribute to improving the capacity of the healthcare workforce to deliver high-quality, inclusive intersex care across diverse healthcare settings.

## Data Availability

The raw data supporting the conclusions of this article will be made available by the authors, without undue reservation.

## References

[1] European Commission. LGBTIQ Equality at a Crossroads – Progress and Challenges. Brussels: European Commission (2024).

[2] Patterson CJ Sepulveda MJ White J. Understanding the Well-Being of LGBTQI+ Populations. Washington, DC: National Academies Press (2021). 33104312

[3] Zeeman L Aranda K. A systematic review of the health and healthcare inequalities for people with intersex variance. Int J Environ Res Public Health. (2020) 17:6533. doi: 10.3390/ijerph17186533, 32911732 PMC7559554

[4] Sherriff N Zeeman L McGlynn N Pinto N Hugendubel K Mirandola M . Co-producing knowledge of lesbian, gay, bisexual, trans and intersex (LGBTI) health-care inequalities via rapid reviews of grey literature in 27 EU member states. Health Expect. (2019) 22:688–700. doi: 10.1111/hex.12934, 31228361 PMC6737757

[5] Lee PA Houk CP Ahmed SF Hughes IA. Consensus statement on management of intersex disorders. Pediatrics. (2006) 118:e488–500. doi: 10.1542/peds.2006-0738, 16882788

[6] Lee PA Nordenström A Houk CP Ahmed SF Auchus R Baratz A . Global disorders of sex development update since 2006: perceptions, approach and care. Horm Res Paediatr. (2006) 85:158–80. doi: 10.1159/000442975, 26820577

[7] Griffiths DA. Shifting syndromes: sex chromosome variations and intersex classifications. Soc Stud Sci. (2018) 48:125–48. doi: 10.1177/0306312718757081, 29424285 PMC5808814

[8] Sandberg DE Gardner M. Differences/disorders of sex development: medical conditions at the intersection of sex and gender. Annu Rev Clin Psychol. (2022) 18:201–31. doi: 10.1146/annurev-clinpsy-081219-101412, 35216524 PMC10170864

[9] Thyen U Lux A Jürgensen M Hiort O Köhler B. Utilization of health care services and satisfaction with care in adults affected by disorders of sex development (DSD). J Gen Intern Med. (2014) 29:752–9. doi: 10.1007/s11606-014-2917-7, 25029980 PMC4124114

[10] Nordenström A Thyen U. Improving the communication of healthcare professionals with affected children and adolescents. Endocr Dev. (2014) 27:113–27. doi: 10.1159/000363636 25247649

[11] Liang JJ Gardner IH Walker JA Safer JD. Observed deficiencies in medical student knowledge of transgender and intersex health. Endocr Pract. (2017) 23:897–906. doi: 10.4158/EP171758.OR, 28534684

[12] DeVita T Bishop C Plankey M. Queering medical education: systematically assessing LGBTQI health competency and implementing reform. Med Educ Online. (2018) 23:1510703. doi: 10.1080/10872981.2018.1510703, 30157712 PMC6116674

[13] Grimstad F Kremen J Streed CG Dalke KB. The health care of adults with differences in sex development or intersex traits is changing: time to prepare clinicians and health systems. LGBT Health. (2021) 8:439–43. doi: 10.1089/lgbt.2021.0018, 34191611

[14] Prandelli M Testoni I. Inside the doctor’s office. Talking about intersex with Italian health professionals. Cult Health Sex. (2021) 23:484–99. doi: 10.1080/13691058.2020.1805641, 32935650

[15] Finlayson C Johnson EK Chen D Fechner PY Hirsch J Rosoklija I . Fertility in individuals with differences in sex development: provider knowledge assessment. J Pediatr Adolesc Gynecol. (2022) 35:558–61. doi: 10.1016/j.jpag.2022.02.004, 35296452 PMC9468186

[16] Atayan AB Huerne K Palmour N Joly Y. Towards equity & inclusion: a critical examination of genetic counselling education on intersex healthcare. BMC Med Educ. (2024) 24:942. doi: 10.1186/s12909-024-05898-x, 39210433 PMC11360692

[17] Donisi V Amaddeo F Zakrzewska K Farinella F Davis R Gios L . Training healthcare professionals in LGBTI cultural competencies: exploratory findings from the Health4LGBTI pilot project. Patient Educ Couns. (2020) 103:978–87. doi: 10.1016/j.pec.2019.12.007, 31866197

[18] Pratt-Chapman ML Eckstrand K Robinson A Beach LB Kamen C Keuroghlian AS . Developing standards for cultural competency training for health care providers to care for lesbian, gay, bisexual, transgender, queer, intersex, and asexual persons: consensus recommendations from a National Panel. LGBT Health. (2022) 9:340–7. doi: 10.1089/lgbt.2021.0464, 35443812 PMC9291720

[19] Buery-Joyner SD Baecher-Lind L Clare CA Hampton BS Moxley MD Ogunyemi D . Educational guidelines for diversity and inclusion: addressing racism and eliminating biases in medical education. Am J Obstet Gynecol. (2023) 228:133–9. doi: 10.1016/j.ajog.2022.09.014, 36113577

[20] Fimognari N Kardol LR O’Shannassy T Sanders KA Smith JT Wyrwoll CS. Inclusion of genital, sexual, and gender diversity in human reproductive teaching: impact on student experience and recommendations for tertiary educators. Adv Physiol Educ. (2024) 48:698–703. doi: 10.1152/advan.00113.2024, 39116390

[21] Saini S MacDonald J Clunie M Slark J Prebble K Paton N . Embedding LGBTQI+ competency into nursing education: formative evaluation of an interdisciplinary project. Nurse Educ Today. (2022) 119:105546. doi: 10.1016/j.nedt.2022.105546, 36155208

[22] Prasad S O’Malley CB DeLeon R Levy AS Griffin DP. Inclusive LGBTQIA+ healthcare: an interprofessional case-based experience for cultural competency awareness. Front Public Health. (2023) 10:993461. doi: 10.3389/fpubh.2022.993461, 36684971 PMC9846843

[23] Bertalan R Lucas-Herald A Kolesinska Z Berra M Cools M Balsamo A . Evaluation of DSD training schools organized by cost action BM1303 “DSDnet”. Orphanet J Rare Dis. (2018) 13:227. doi: 10.1186/s13023-018-0967-3, 30563557 PMC6299629

[24] Kranenburg L Reerds STH Cools M Alderson J Muscarella M Grijpink K . Global application of assessment of competencies of paediatric endocrinology fellows in the management of differences of sex development (DSD) using the ESPE e-learning.Org portal. Med Sci Educ. (2016) 26:679–89. doi: 10.1007/s40670-016-0333-9

[25] Kranenburg LJC Reerds STH Cools M Alderson J Muscarella M Magrite E . Global application of the assessment of communication skills of paediatric endocrinology fellows in the management of differences in sex development using the ESPE e-learning.Org portal. Horm Res Paediatr. (2017) 88:127–39. doi: 10.1159/000475992, 28689203

[26] Gupta A Lockeman K Edwards C. Increasing knowledge and self-efficacy on differences in sex development (DSD): a team-based learning activity for pediatric residents. MedEdPORTAL. (2021) 17:11105. doi: 10.15766/mep_2374-8265.11105, 33644305 PMC7901252

[27] Schmidt HG Rotgans JI Yew EH. The process of problem-based learning: what works and why. Med Educ. (2011) 45:792–806. doi: 10.1111/j.1365-2923.2011.04035.x, 21752076

[28] Favazzi UM Marconi M Carbone P Guerrera D Ruocco A Manoli M . Evaluating the impact of distance learning on gender-affirming healthcare competence: knowledge acquisition and satisfaction among healthcare professionals in Italy. Front Public Health. (2024) 12:1393188. doi: 10.3389/fpubh.2024.1393188, 38903566 PMC11187281

[29] Cruciani G Quintigliano M Mezzalira S Scandurra C Carone N. Attitudes and knowledge of mental health practitioners towards LGBTQ+ patients: a mixed-method systematic review. Clin Psychol Rev. (2024) 113:102488. doi: 10.1016/j.cpr.2024.102488, 39168053

[30] Godø TB Bjørndal Å Fluge IM Johannessen R Lavdas M. Personality traits, ideology, and attitudes toward LGBT people: a scoping review. J Homosex. (2024) 72:1–20. doi: 10.1080/00918369.2024.2344015, 38656199

[31] Ebbinghaus H. Memory: a contribution to experimental psychology. Ann Neurosci. (2013) 20:155–6. doi: 10.5214/ans.0972.7531.200408, 25206041 PMC4117135

[32] Custers EJFM. Long-term retention of basic science knowledge: a review study. Adv Health Sci Educ. (2010) 15:109–28. doi: 10.1007/s10459-008-9101-y, 18274876

[33] Albert FA Seidu A-A Mason HM Anderson E Alele FO Heggarty P . A systematic review of medical practitioners’ retention and application of basic sciences to clinical practice. BMC Med Educ. (2024) 24:997. doi: 10.1186/s12909-024-05952-8, 39272053 PMC11396528

