# Supplementary material for: Intersex health training is associated with sustained competency gains in the healthcare workforce
Source: Front Public Health. 2026 Jun 29;14:1809092. doi: 10.3389/fpubh.2026.1809092 (PMC13357704; doi:10.3389/fpubh.2026.1809092)
Supplement: Supplementary file 1 [file Data_Sheet_1.docx]

Supplementary Material

# Supplementary Figures and Tables

**eTable 1.** Detailed overview of the training modules and core content

**eTable 2.** *Knowledge Assessment Test*: items used to evaluate Learning Outcomes

**eTable 3.** Exploratory Factor Analysis of the Attitudes and Skills Questionnaire (ASQ) across time points

**eTable 4.** Participant satisfaction at immediate post-training (T1) (n = 6,319)

**eTable 5.** Characteristics of participants completing the 6-month follow-up assessment (T2)

**eTable 6.** Self-reported attitudes toward intersex care at baseline (T0), immediate post-training (T1) and 6-month follow-up (T2) (n = 1,036)

**eTable 7.** Self-reported skills in intersex care at baseline (T0), immediate post-training (T1) and at 6-month follow-up (T2) (n = 1,036)

**eTable 8.** Knowledge levels on intersex care by learning objective at baseline (T0), immediate post-training (T1), and at 6-month follow-up (T2) (n = 1,036)

**eFigure 1.** Self-reported attitudes, self-reported skills, and knowledge levels of participants at baseline (T0) and immediately after training (T1) (panels a–c; n = 6,332), and at the 6-month follow-up (T2) (panels d–f; n = 1,036).

**eTable 1. Detailed overview of the training modules and core content**

| Learning Objective (LO) | Module Title | Core Content |
| --- | --- | --- |
| LO1 | **Components of sexual identity and its biological foundations** | Components of sexual identity (sex assigned at birth, gender identity, gender expression, sexual orientation); binary and non-binary identities; biological processes of sex differentiation (chromosomal, gonadal, phenotypic); terminology and definitions related to intersex variations |
| LO2 | **Psychological and medical aspects of intersex care across the lifespan** | Clinical presentation and diagnostic pathways; gender assignment considerations; hormonal aspects and medical management; fertility implications; psychological support; impact of stigma and minority stress; longitudinal and multidisciplinary care |
| LO3 | **The health provider–patient relationship and approaches to family support** | Communication of diagnosis and care pathways; roles and responsibilities of healthcare professionals; multidisciplinary team approach; family support strategies; shared decision-making; patient-centered care and psychoeducation |
| LO4 | **Legal aspects related to intersex variations** | Italian legal framework; sex assignment at birth and legal registration; legal gender recognition procedures; constitutional principles (dignity, equality, right to health, self-determination); emerging legal and human rights perspectives |
| LO5 | **Ethical considerations in the care of individuals with intersex variations** | Bioethical principles (autonomy, beneficence, non-maleficence, justice); informed consent and right to information; protection of bodily integrity; ethical decision-making in clinical practice; specific considerations in minors |

**eTable 2. *Knowledge Assessment Test*: items used to evaluate Learning Outcomes**

| **Question Code** | **Question Text** |
| --- | --- |
| **Q1** | The four components of sexual identity are [*Choose one*]  A. Assigned sex at birth, registered sex, gender identity, sexual orientation  B. Gender identity, sexual orientation, gender incongruence, assigned sex at birth  C. Gender identity, assigned sex at birth, gender expression, sexual orientation ✅  D. Gender identity, assigned sex at birth, gender expression, intersex conditions |
| **Q2** | How is gender identity defined? [*Choose one*]  A. Physical and/or romantic attraction to others, based on one’s own and their partner’s gender identity  B. Classification as male, female, or intersex based on physical characteristics  C. Deep and personal sense of belonging to a gender (male, female, or another) ✅  D. The way a person expresses their gender through daily behaviour |
| **Q3** | Which of the following is a key medical consideration in the care of intersex individuals at birth? [*Choose one*]  A. Prompt management of any urgent clinical issues ✅  B. Immediate initiation of gender-affirming hormone therapy  C. Determination of sexual orientation  D. Assignment of gender identity based solely on assigned sex at birth |
| **Q4** | According to international expert consensus recommendations, which of the following statements is *incorrect* regarding the care of intersex individuals? [*Choose one*]  A. Professionals and parents should communicate with children in developmentally appropriate ways  B. Professionals should never intervene in communication between parents and children to avoid influencing gender identity ✅  C. Professionals and parents should support gender identity exploration throughout life  D. Efforts should be made to promote well-being and reduce stigma associated with intersex conditions |
| **Q5** | In the care of intersex individuals, the ideal healthcare team [*Choose one*]  A. Is not associated with increased perceived quality of care  B. Should include various specialists tailored to the individual's needs ✅  C. Does not require interdisciplinary collaboration  D. Plays no central role in improving access to care. |
| **Q6** | Which of the following statements about supporting gender identity exploration is *incorrect*? [*Choose one*]  A. Intersex conditions should be understood as natural human variations  B. In cases of gender incongruence, referral to specialized services should be considered  C. Non-binary understandings of sex and gender should be supported  D. Gender questioning should be discouraged ✅ |
| **Q7** | Under Italian law, the sex–gender binary is [*Choose one*]  A. Legally established, but can be set aside if parents or the individual prefer a neutral identity  B. Legally established, but may be waived if the individual requests a neutral identity in the case of intersex variation  C. Legally established, though neutral naming and sex markers are freely available throughout life  D. Explicitly mandated by law, requiring names consistent with sex assigned at birth ✅ |
| **Q8** | Under Italian law, intersex conditions are [*Choose one*]  A. Explicitly regulated through detailed provisions recognizing a “third sex”  B. Not specifically regulated; the legal system refers almost exclusively to sex, not gender ✅  C. Recognized and protected, with enforcement mechanisms against noncompliance  D. Strongly safeguarded through constitutional and international law guarantees |
| **Q9** | According to Italian Law 219/2017, which of the following is not a feature of how consent is regulated for minors? [*Choose one*]  A. Right to access information  B. Physician’s judgment always prevails, even over parental consent ✅  C. Support for improving minors' understanding and decision-making capacity  D. Consent expressed by parents in the minor’s best interest |
| **Q10** | Which of the following is not a guiding principle in medical decision-making for minors with intersex variations? [*Choose one*]  A. Minor’s right to information  B. The physician has sole authority to decide in the minor’s best interest, even against parental consent ✅  C. Establishing a caring relationship involving minors and parents  D. Minor’s involvement in decisions, according to age and maturity |

This table presents the multiple-choice questions included in the Knowledge Assessment Test, designed to evaluate participants’ learning outcomes. The correct answer for each item is indicated with a checkmark (✅). The questions are mapped to the course learning objectives (LOs), representing the intended learning outcomes of the training program.

**eTable 3. Exploratory Factor Analysis of the Attitudes and Skills Questionnaire (ASQ) across time points**

| EFA summary |
| --- |
| \| **Time point** \| **Factor** \| **Eigenvalue** \| **Variance explained (%)** \| **Cronbach’s α** \| \| --- \| --- \| --- \| --- \| --- \| \| T0 \| Skills \| 3.447 \| 26.9 \| 0.850 \| \| T0 \| Attitudes \| 3.121 \| 25.2 \| 0.839 \| \| T1 \| Skills \| 5.362 \| 33.8 \| 0.902 \| \| T1 \| Attitudes \| 2.469 \| 31.9 \| 0.902 \| \| T2 \| Skills \| 4.630 \| 32.4 \| 0.892 \| \| T2 \| Attitudes \| 2.568 \| 26.6 \| 0.841 \| |
| Range of factor loadings by item group |
| \| **Item group** \| **T0 loading range** \| **T1 loading range** \| **T2 loading range** \| \| --- \| --- \| --- \| --- \| \| Skills items on Skills factor \| 0.567–0.797 \| 0.757–0.821 \| 0.721–0.830 \| \| Attitudes items on Attitudes factor \| 0.336–0.886 \| 0.399–0.958 \| 0.280–0.920 \| |

Exploratory Factor Analysis of the ASQ across time points (T0, T1, T2). Eigenvalues, percentage of explained variance, and Cronbach’s alpha coefficients are reported for each factor. All items loaded on their intended factor with loadings ≥0.40 and no substantial cross-loadings were observed.

**eTable 4. Participant satisfaction at immediate post-training (T1)** (n = 6,319)

| **Statement** | **Satisfaction score, mean (SD)** |
| --- | --- |
| **Methodology** |  |
| The course objectives were clearly defined | 4.55 (0.65) |
| The course content was consistent with the stated objectives | 4.55 (0.65) |
| The teaching methodology was effective | 4.59 (0.62) |
| The overall organization (course structure, scheduling, interim and final assessments) was satisfactory | 4.57 (0.63) |
| The test questions were sufficiently clear | 4.41 (0.78) |
| The time allocated for completing the tests was adequate | 4.42 (0.74) |
| The quality of tutoring for this distance learning (Formazione a Distanza [FAD]) event was satisfactory | 4.46 (0.71) |
| **Platform** |  |
| The quality of technical support for this FAD event was satisfactory | 4.58 (0.66) |
| The functionality of the electronic platform used for this FAD event was adequate | 4.45 (0.73) |
| The access procedures for the platform were quick and simple | 4.57 (0.68) |
| **Contents** |  |
| The level of instruction was appropriate to my prior knowledge | 4.44 (0.74) |
| I acquired new concepts | 4.38 (0.73) |
| I developed new skills | 4.57 (0.66) |
| I am able to apply what I learned in this course to my professional practice | 4.35 (0.79) |
| The documentation provided was sufficient to acquire the necessary information | 4.11 (0.91) |
| The quality of the documentation provided was appropriate | 4.54 (0.67) |
| The documentation provided was up-to-date with the most recent literature | 4.55 (0.66) |
| Consulting the Participant’s Guide was helpful in navigating the learning process | 4.50 (0.71) |

The table reports mean item-level scores among participants (based on a 5-point Likert scale, with higher scores indicating higher satisfaction) and corresponding standard deviations (SDs) for the subset of participants who completed the Satisfaction questionnaire at immediate post-training (T1) (n = 6,319). FAD, Formazione a Distanza, an Italian term for distance learning.

**eTable 5. Characteristics of participants completing the 6-month follow-up assessment (T2)**

| **Characteristic** | **Participants, No. (%) (n = 1,036)** |
| --- | --- |
| **Gender** |  |
| Male | 286 (27.6) |
| Female | 750 (72.4) |
| **Age (years)** |  |
| Up to 35 | 112 (10.8) |
| 36 to 45 | 208 (20.1) |
| 46 to 55 | 257 (24.8) |
| Over 55 | 459 (44.3) |
| **Italian Region Area** |  |
| Northwest | 313 (30.2) |
| Northeast | 136 (13.1) |
| Center | 299 (28.9) |
| South | 174 (16.8) |
| Islands | 113 (10.9) |
| Foreign | 1 (0.1) |
| **Health professions** |  |
| Healthcare Assistant | 37 (3.6) |
| Midwife | 72 (6.9) |
| Physician | 407 (39.3) |
| Psychologist | 520 (50.2) |

**eTable 6. Self-reported attitudes toward intersex care at baseline (T0), immediate post-training (T1) and 6-month follow-up (T2) (n = 1,036)**

|  | Mean (SD) | | |  |  |  |  |
| --- | --- | --- | --- | --- | --- | --- | --- |
| Statement | T0 | T1 | T2 | Mean difference T2-T0 | p value | Mean difference T2-T1 | p value |
| Biological factors significantly influence the development of gender differences | 2.32 (1.22) | 3.15 (1.30) | 2.54 (1.24) | +0.22 (1.42) | <.001 | -0.61 (1.48) | <.001 |
| Psychological support should be provided to intersex individuals during the coming out process | 3.77 (1.29) | 4.25 (1.11) | 4.13 (1.20) | +0.36 (1.40) | <.001 | -0.12 (1.26) | <.005 |
| Support resources should be made available to families of individuals diagnosed with an intersex variation | 3.79 (1.26) | 4.33 (1.07) | 4.13 (1.20) | +0.34 (1.38) | <.001 | -0.20 (1.26) | <.001 |
| Comprehensive legal information should be made available regarding intersex variations | 3.79 (1.23) | 4.21 (1.11) | 4.14 (1.16) | +0.35 (1.33) | <.001 | -0.07 (1.25) | .112 |
| Bioethical principles relevant to intersex variations must be taken into account | 3.39 (1.40) | 4.18 (1.14) | 3.73 (1.32) | +0.34 (1.55) | <.001 | -0.45 (1.39) | <.001 |

The table reports mean item-level scores among participants (based on a 5-point Likert scale, with higher scores indicating more positive attitudes toward intersex care) and corresponding standard deviations (SDs) for the subset of participants who completed the baseline (T0), immediate post-training (T1) and 6-month follow up (T2) assessments (n = 1,036). Change scores represent the average per-item difference, calculated as the arithmetic difference between T2 and T0, and between T2 and T1. All p-values are from paired t-tests comparing mean scores between T0 and T1 and between T2 and T1. All statements were preceded in the questionnaire by the prompt: “*With regard to the care of intersex individuals, I believe that*…”

**eTable 7. Self-reported skills in intersex care at baseline (T0), immediate post-training (T1) and at 6-month follow-up (T2)** (n = 1,036)

|  | Mean (SD) | | |  |  |  |  |
| --- | --- | --- | --- | --- | --- | --- | --- |
| Statement | T0 | T1 | T2 | Mean difference T2-T0 | p value | Mean difference T2-T1 | p value |
| Describe the components of sexual identity and their biological foundations | 2.50 (1.16) | 3.94 (0.71) | 3.50 (0.92) | +1.00 (1.27) | <.001 | -0.44 (0.91) | <.001 |
| Identify relevant psychological and medical factors in the care of intersex individuals | 2.46 (1.14) | 3.96 (0.69) | 3.57 (0.88) | +1.11 (1.27) | <.001 | -0.39 (0.87) | <.001 |
| Identify tools and resources to support families of intersex individuals | 2.58 (1.19) | 4.05 (0.69) | 3.73 (0.88) | +1.15 (1.26) | <.001 | -0.32 (0.88) | <.001 |
| Define the roles and responsibilities of healthcare professionals in relation to service users | 2.65 (1.18) | 4.03 (0.66) | 3.74 (0.87) | +1.09 (1.30) | <.001 | -0.29 (0.87) | <.001 |
| Identify legal frameworks associated with intersex conditions | 1.94 (0.94) | 3.55 (0.90) | 2.94 (1.12) | +1.00 (1.33) | <.001 | -0.61 (1.15) | <.001 |
| Identify key bioethical issues related to intersex conditions | 2.06 (1.03) | 3.91 (0.72) | 3.30 (1.04) | +1.24 (1.27) | <.001 | -0.61 (1.05) | <.001 |

The table reports mean item-level scores among participants (based on a 5-point Likert scale, with higher scores indicating improved skills in intersex care) and corresponding standard deviations (SDs) for the subset of participants who completed both the baseline (T0), immediate post-training (T1) and 6-month follow up (T2) assessments (n = 1,036). Change scores represent the average per-item difference, calculated as the arithmetic difference between T0 and T1 and between T2 and T1. All p-values are from paired t-tests comparing mean scores between T0 and T1. All statements were preceded in the questionnaire by the prompt: “*With regard to the care of intersex individuals, if given the opportunity, I would be able to*…”

**eTable 8.** **Knowledge levels on intersex care by learning objective at baseline (T0), immediate post-training (T1), and at 6-month follow-up (T2)** (n = 1,036)

|  |  | Correct answers, No. (%) | | |  |  |  |  |
| --- | --- | --- | --- | --- | --- | --- | --- | --- |
| Learning Objective (LO) | **Quest.** | T0 | T1 | T2 | T2-T0, No. (pp) | p value | T2-T1, No. (pp) | p value |
| LO1: Components of sexual identity and its biological foundations | Q1 | 559 (54.0) | 810 (78.2) | 718 (69.3) | +159 (15.3) | <.001 | -92 (8.9) | <.001 |
|  | Q2 | 778 (75.1) | 860 (83.0) | 843 (81.4) | +65 (6.3) | <.005 | -17 (1.6) | .065 |
| LO2: Psychological and medical aspects of intersex care across the lifespan | Q3 | 762 (73.6) | 905 (87.4) | 855 (82.5) | +93 (8.9) | <.001 | -50 (4.9) | <.001 |
|  | Q4 | 635 (61.3) | 800 (77.2) | 710 (68.5) | +75 (7.2) | <.005 | -90 (8.7) | <.001 |
| LO3: The healthcare provider–patient relationship and approaches to family support | Q5 | 986 (95.2) | 997 (96.2) | 1006 (97.1) | +20 (1.9) | 0.735 | +9 (0.9) | .243 |
|  | Q6 | 679 (65.5) | 813 (78.5) | 739 (71.3) | +60 (5.8) | 0.022 | -74 (7.2) | <.001 |
| LO4: Legal aspects related to intersex variations | Q7 | 325 (31.4) | 690 (66.6) | 452 (43.6) | +127 (12.2) | <.001 | -238 (23.0) | <.001 |
|  | Q8 | 691 (66.7) | 787 (76.0) | 760 (73.4) | +69 (6.7) | <.005 | -27 (2.6) | <.005 |
| LO5: Ethical considerations in the care of individuals with intersex variations | Q9 | 764 (73.7) | 887 (85.6) | 820 (79.2) | +56 (5.5) | 0.025 | -67 (6.4) | <.001 |
|  | Q10 | 886 (85.5) | 936 (90.3) | 932 (90.0) | +46 (4.5) | <.005 | -4 (0.3) | .246 |

The table reports item-level data (*Knowledge Assessment Test*, Q1–Q10) on the number and percentage of correct answers for the subset of participants who completed the baseline (T0), immediate post-training (T1) and 6-month follow up (T2) assessments (n = 1,036), grouped by Learning Objectives (LOs). Blank responses were automatically coded as incorrect. Changes (T2–T0 and T2-T1) represent the absolute difference in correct response rates between time points, expressed in percentage points (pp). All p-values are from McNemar’s test for paired proportions, used to assess the statistical significance of changes in correct responses between T2 and T0 and between T2 and T1.

**eFigure 1. Self-reported attitudes, self-reported skills, and knowledge levels of participants at baseline (T0) and immediately after training (T1)** (panels a–c; n=6,332)**, and at the 6-month follow-up (T2)** (panels d–f; n=1,036).


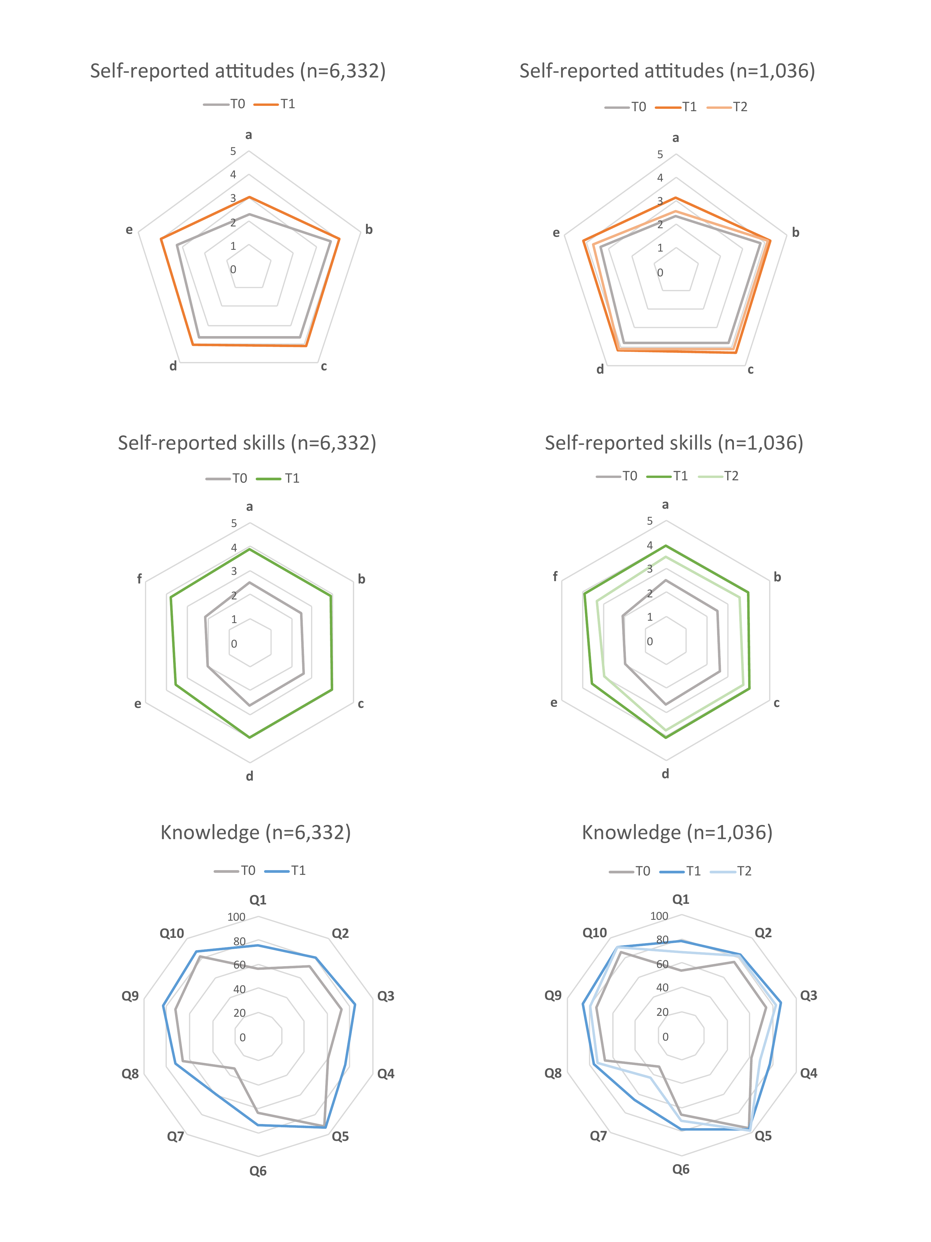


Radar-plot vertices correspond to attitudes and skills items (Attitudes and Skills Questionnaire; ASQ) and to knowledge assessment items used to evaluate Learning Outcomes (Q1–Q10, Knowledge Assessment Test; KAT). Lines represent mean item-level scores among participants. The figure provides a visual summary of longitudinal patterns across domains at baseline (T0), post-training (T1), and six-month follow-up (T2).

**
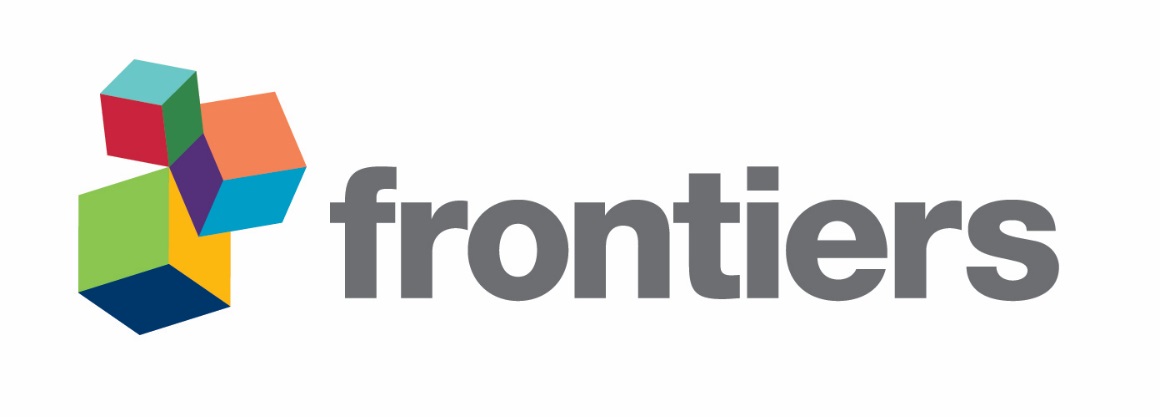
**
